# A questionnaire to identify patellofemoral pain in the community: an exploration of measurement properties

**DOI:** 10.1186/s12891-016-1097-5

**Published:** 2016-05-31

**Authors:** Paola Dey, Michael Callaghan, Neil Cook, Ruth Sephton, Chris Sutton, Elaine Hough, Jonathan James, Rukhtam Saqib, James Selfe

**Affiliations:** College of Clinical and Biomedical Sciences, University of Central Lancashire, Preston, PR1 2HE UK; Department of Health Professions, Manchester Metropolitan University, Manchester, M15 6GX UK; College of Health and Wellbeing, University of Central Lancashire, Preston, PR1 2HE UK; 5 Boroughs Partnership NHS Foundation Trust, St Helens Hospital, Marshalls Cross Road, St Helens, WA9 3DA UK; Stepping Hill Hospital, Stockport Foundation NHS Trust, Poplar Grove, Hazel Grove, Stockport, Cheshire SK2 7JE UK

**Keywords:** Patellofemoral pain, Diagnosis, Differential, Validation studies, Reliability, Sensitivity and specificity

## Abstract

**Background:**

Community-based studies of patellofemoral pain (PFP) need a questionnaire tool that discriminates between those with and those without the condition. To overcome these issues, we have designed a self-report questionnaire which aims to identify people with PFP in the community.

**Methods:**

Study designs: comparative study and cross-sectional study.

Study population: comparative study: PFP patients, soft-tissue injury patients and adults without knee problems. Cross-sectional study: adults attending a science festival.

Intervention: comparative study participants completed the questionnaire at baseline and two weeks later. Cross-sectional study participants completed the questionnaire once.

The optimal scoring system and threshold was explored using receiver operating characteristic curves, test-retest reliability using Cohen’s kappa and measurement error using Bland-Altman plots and standard error of measurement. Known-group validity was explored by comparing PFP prevalence between genders and age groups.

**Results:**

Eighty-four participants were recruited to the comparative study. The receiver operating characteristic curves suggested limiting the questionnaire to the clinical features and knee pain map sections (AUC 0.97 95 % CI 0.94 to 1.00). This combination had high sensitivity and specificity (over 90 %). Measurement error was less than the mean difference between the groups. Test–retest reliability estimates suggest good agreement (*N* = 51, *k* = 0.74, 95 % CI 0.52–0.91). The cross-sectional study (*N* = 110) showed expected differences between genders and age groups but these were not statistically significant.

**Conclusion:**

A shortened version of the questionnaire, based on clinical features and a knee pain map, has good measurement properties. Further work is needed to validate the questionnaire in community samples.

**Electronic supplementary material:**

The online version of this article (doi:10.1186/s12891-016-1097-5) contains supplementary material, which is available to authorized users.

## Background

Patellofemoral pain (PFP) is generally considered a commonly occurring self-limiting condition. However, this assumption has been challenged. A review by Callaghan and Selfe suggests the prevalence of PFP varies between 3 % and 40 %, but most of the studies reviewed were undertaken in military or sporting cohorts [1]. More recent studies in general populations have been limited to schoolchildren [2, 3]. There are also growing concerns about the long-term outcomes of PFP such as an increased risk of patellofemoral osteoarthritis [4, 5], and a recent systematic review of studies investigating this association has highlighted the need for further research [6]. Such concerns about long-term harm suggest a need to consider preventative strategies to reduce the risk of PFP. However, to date, studies of PFP risk factors have tended to focus on biomechanical factors rather than on factors such as footwear, obesity or psychosocial factors, which may be more amenable to large-scale preventative action [7]. Investigation of the prevalence and incidence of PFP, its natural history and modifiable risk factors requires large-scale, population-based studies. However there is no specific measurement instrument to identify those who suffer from PFP in the community. There are self-report measures of PFP outcome and measures to identify those with a range of patellofemoral disorders [8–11], but these are not designed to specifically discriminate between those that have PFP and those that do not in general populations. Knee pain maps alone might facilitate location of the pain, but cannot necessarily exclude other conditions [12]. Previous studies in sporting and military populations have used clinical examination directly or data from medical records [7]. Population-based studies in adolescents have also relied on clinical assessment to identify those with PFP [2, 3]. One of the major drawbacks of clinical assessment in large cohort studies is the cost and inconvenience to participants. Furthermore, in clinical practice, PFP tends to be diagnosed by excluding other knee conditions first [13, 14]. Retrospective examination of routine records also has drawbacks due to problems of correctly identifying the condition using the International Classification of Disease because there is no specific code for PFP. A study in primary care in England has suggested probable under-ascertainment because PFP may be recorded under general terms such as ‘anterior knee pain’ or ‘knee pain’ [15].

To overcome these issues, we have designed a self-report questionnaire (SNAPPS- Survey instrument for Natural history, Aetiology and Prevalence of Patellofemoral pain Studies) which aims to identify people with PFP in the community.

## Methods

### Development of the questionnaire

PFP has been characterised by anterior knee or retropatellar pain, often bilateral, of insidious onset present for at least a month and associated with pain or difficulty with prolonged sitting or activities which load the patellofemoral joint, e.g., ascending or descending stairs, running and squatting [13, 16, 17]. The presence of previous knee surgery, patellofemoral instability and/or knee joint effusion is less likely in PFP, as defined above. To reflect this, our questionnaire consists of four sections. The purpose of the first section is to identify those with knee pain. It uses the question, ‘have you had pain or problems **in the last year** in or around the knee?,’ adapted from the KNEST questionnaire [18]. If present, the subject completes the remaining three sections of the questionnaire. Section two covers clinical features of the knee problem (see Table 1); section three covers pain or difficulty on a number of activities commonly associated with knee problems (see Table 1). The purpose of the last section is to identify that the location of the pain is the patella in order to discriminate between those with PFP and those with other non-specific knee pain. The Knee Pain Map, developed and validated by Elson et al. [12], is used to help respondents locate the origin of their pain [used with permission from David Elson]. The early development of the SNAPPS questionnaire is described elsewhere [14] but, essentially, it has been piloted twice in a total of 20 patients with PFPS and other soft-tissue knee problems attending physiotherapy clinics. Participants made comments on the structure of the questions and whether they covered relevant issues. They also completed the questionnaire allowing the researchers to test the ease of completion and explore scoring [14]. These early attempts highlighted that pain and difficulty while performing activities historically considered to be associated with PFP (e.g. stairs ascent, descent, prolonged sitting) did not discriminate between those with PFP and those with other soft-tissue injury [14]. Following discussion with experts in the field, the most recent version of the questionnaire (SNAPPS IV), includes activities such as pain or difficulty on standing, walking on an uneven surface, walking up slopes and walking on level surfaces. These activities are not associated with loading of the patellofemoral joint but may be associated with soft-tissue injury. We report on the further development and testing of the measurement properties of this version of the questionnaire in a clinical and general population. This consists of the findings of two studies. The first study is an examination of the discriminatory ability and test-retest reliability of SNAPPS IV in a healthy non-injured group and two patient groups whose knee problem was known. This study was used to confirm the best scoring system for this questionnaire. The second study tested the questionnaire, and the optimal scoring system from study one, in a general population of adults in whom the presence or absence of knee problems was unknown. The objective of this second study was to investigate known-group validity. That is, that in classifying PFP, the characteristics of those classified would be in line with studies in PFP. It was hypothesised that the questionnaire would identify approximately twice as many women as men with probable PFP [19], and also that PFP would be more frequent in those under 40 years of age [20].

**Table 1:** Participant characteristics and item scores for the two studies

Groups	Study 1-baseline			Study 2
	PFP^a^ (*N* = 26)	Soft-tissue (*N* = 30)	Healthy (*N* = 28)	(*N* = 110)
Patient characteristics				
Mean age (SD) in years	27 (7.8)	27 (6.8)	28 (5.9)	30 (9.9)
Gender–female	10 (39 %)	14 (47 %)	14 (50 %)	58 (53 %)^b^
Mean body mass index (SD)	26 (6.6)	26 (5.0)^b^	24 (3.5)	25 (4.4)^c^
Section 1:				
‘Have you **ever** been to a doctor because of knee problems?’ Yes	22 (85 %)	30 (100 %)	5 (18 %)	36 (33 %)
‘Have you had pain or problems **in the last year** in or around the knee?’ Yes				
	25 (96 %)	28 (93 %)	2 (7 %)	38 (35 %)
Section 2: Clinical features				
In which knee have you had pain or problems? Both	16 (62 %)	5 (17 %)	1	17 (15 %)
Have you had surgery to your knee? No	25 (96 %)	27 (90 %)	2	4 (4 %)
Have you ever had a knee cap that has gone out of joint (dislocated)? No	25 (96 %)	26 (90 %)^b^	2	2 (2 %)
Since starting with your knee problem, does your knee **ever swell up**? No	11 (42 %)	4 (13 %)	2	15 (14 %)
Have you had pain and discomfort for **more than one month**? Yes	22 (85 %)	13 (43 %)	1	16 (15 %)
Thinking about your **right (left)** knee, what do you consider is your **main problem** with your knee? Pain and discomfort	25 (96 %)	23 (77 %)	2	34 (31 %)
Thinking about your **right (left)** knee, did your current knee problem come on? Gradually	21 (81 %)	4 (13 %)	0	18 (16 %)
Total mean (SD) score for section 2	5.5 (0.91)^d^	3.4 (1.22)^d^	0.36 (1.31)^d^	1.6 (2.31)
Section 3: activity related pain				
Because of your knee problems would you suffer from pain or difficulty with:				
Sitting for a long time Yes	16 (62 %)	17 (57 %)	1	11 (10 %)
Going upstairs Yes	21 (81 %)	29 (97 %)	1	19 (17 %)
Going downstairs Yes	17 (65 %)	24 (80 %)	1	12 (11 %)
Squatting Yes	20 (77 %)	30 (100 %)	1	20 (18 %)
Standing for long periods No	12 (46 %)	4 (13 %)	2	22 (20 %)
Walking on a level surface No	13 (50 %)	6 (21 %)^b^	2	29 (26 %)
Getting out of a chair Yes	23 (89 %)	20 (67 %)	0	6 (5 %)
Kneeling Yes	21 (81 %)	29 (97 %)	0	26 (24 %)
Walking on uneven surfaces No	7 (27 %)	4 (13 %)	1	24 (22 %)
Walking down slopes Yes	17 (65 %)	24 (80 %)	1	20 (18 %)
Walking up slopes No	7 (27 %)	3 (10 %)	1	23 (21 %)
Hopping Yes	17 (65 %)	29 (97 %)	2	14 (13 %)
Jumping Yes	18 (69 %)	27 (90 %)	2	16 (15 %)
Running Yes	19 (73 %)	39 (100 %)	2	20 (18 %)
Total mean (SD) score for section 3	9.2 (1.43)	8.8 (1.80)	0.61 (2.33)^e^	6.9 (2.40)
Section 4: knee pain map				
Total mean (SD) score for section 4	2.3 (1.40)^d^	0.6 (0.73)^d^	0.0 (0.00)^d^	0.62 (1.34)
Total mean (SD) score				
All sections combined	16.6 (2.73) ^d^	13.2 (1.67)^d^	1.0 (3.54)^d^	-----------
Section 2 and 4 combined	7.8 (1.74) ^d^	4.0 (1.50)^d^	0.4 (1.31)^d^	2.2 (3.38)

^a^diagnostic criteria agreed with recruiting physiotherapist

^b^1 missing value ^c^4 missing values

^d^Significantly different from either group *P* < 0.001

eSignificantly different from both the other groups *P* < 0.001

Two participants in the healthy group responded that they had knee pain and completed the sections 2, 3, 4; Participants in the healthy group who responded they did not have a knee problem (and did not complete any other sections) were given a score of 0 for each section and a total score of 0 (see box)

### Study one

Three groups of participants, aged between 18 and 40 years, were recruited from three different settings. The first group were participants with a recent clinical diagnosis of PFP who were recruited consecutively, when the recruiting clinician was available, from an NHS outpatient clinic prior to commencement of treatment: these participants are referred to as the ‘PFP group’. The working definition of PFP was “clinical presentation of knee pain related to changes in the patellofemoral joint…. a gradual onset of pain with none of the features associated with other knee diseases or trauma [16, 17].” Patients were included if all or most of the following had been recorded on routine examination: no swelling, pain on palpation of lateral or medial patellar facets or on manual compression test and pain on ascending or descending stairs, squatting, kneeling or prolonged sitting. If an x-ray had been taken previously, there should also be no radiographic evidence of patellofemoral osteoarthritis, but it was not necessary for an x-ray to have been taken. These criteria were agreed with the recruiting physiotherapists. The second group were participants with a recent diagnosis of soft-tissue injury including ligamentous or meniscal injury and who had no history of PFP; these participants were recruited consecutively, when the recruiting clinician was available, from an NHS outpatient setting in another hospital: these participants are referred to as the ‘soft-tissue group’. In each NHS setting, eligibility was assessed by experienced musculoskeletal therapists. The third group consisted of volunteers with no known history of a clinically diagnosed knee problem recruited from University staff and students through local advertisement: these participants are referred to as the ‘healthy knee group’. Those with known osteoarthritis, neuromuscular disorders or gait pathologies were excluded from the study. For all participants, the baseline questionnaire was self-completed after inclusion and exclusion criteria were applied. The follow-up questionnaire was administered two weeks later by post before treatment to PFP and soft-tissue injury participants and face-to-face (but self-completed) for the healthy knee participants. Participants were recruited between February 2012 and October 2013. Ethical approval for the study was obtained from the National Research Ethics Committee (11/NW/0409) and University of Central Lancashire BUSH ethics committee. Informed written consent was obtained.

### Study two

The purpose of this study was to explore the distribution of scores and known group validity in a sample of the general population. Participants were drawn from a convenience sample of adults attending a university science fair who self-completed the questionnaire on one occasion. The sample included university staff and members of the public. Further details are available in Selfe et al. 2015 [21]. We expected that, among participants meeting threshold scores for PFP, there would be at least twice as many females than males and that the distribution of genders would be different between those with knee pain who met the criteria for PFP and those who did not. We also expected that other types of knee pain would be more common in adults over 40 years of age compared to those 18 to 39 years of age. University ethical committee approval for data collection at the Science Festival was obtained.

### Analysis

The method for scoring of items in the questionnaire is shown in the Box.

**Table:** 

BOX: Scoring of the questionnaire Section 1: Participants were first asked ‘Have you had pain or problems **in the last year** in or around the knee?’ if they answered yes, they went onto complete section 2 to 4. Participants who did not have a knee problem (and did not complete any other sections) were given a score of 0 for each section and a total score of 0. Scoring of sections Section 2: A score of one was given for each positive response to a clinical feature indicative of PFP: gradual onset, bilateral problem, pain for one month or more, main problem pain and discomfort. A score of one was given for a negative response to features not indicative of PFP: swelling, previous surgery, dislocation. In the presence of bilateral pain, a maximum score of 1 was given for each clinical feature. Scores were totalled. The minimum score was 0 and the maximum score was 7 for this question. Participants who did not have a knee problem (and did not complete any other sections) were given a score of 0. Section 3: Negative responses to questions relating to standing for long periods, walking up slopes, walking on uneven surfaces and walking on level surfaces were given a score of 1. Positive responses to all other activities were given a score of 1. In the presence of bilateral pain, a maximum score of 1 was given for each activity. Scores were totalled. The minimum score was 0 and the maximum score was 14. Participants who did not have a knee problem (and did not complete any other sections) were given a score of 0. Section 4: A score of 1 was given for each region on the knee pain map which covered the patellar (i.e., medial patella, lateral patella and patella tendon) to give a total score of 3 for each knee. The minimum score was 0 and the maximum score was 6. Finalised total score for questionnaire In the finalised scoring of the questionnaires only the sum scores for sections 2 and 4 were included. The sums of these sections were added together to give a total score. The total score for the questionnaire was between 0 and 13. Participants with knee problems who had a score of 6 or more were considered to have PFP.	

### Study one

For each item on the questionnaire in study one, we report the missing values and the distribution of responses. Inter-item correlations were examined for section 2 and 3. Inter-item correlations measure whether a question is related to another question within a domain, a correlation above 0.7 would suggest that both are measuring similar concepts and a decision can be made to remove one of the questions [22]. Differences in the distribution of mean scores for each section, combination of sections and the total score for all sections combined were explored between the three groups using analysis of variance, with Tukey b or Games-Howell post hoc tests, as appropriate. Differences between groups were also explored using various methods to score the sections such as limiting section 3 to those items not associated with PFP (e.g. pain or difficulty on standing, walking on an uneven surface, walking up slopes and walking on level surfaces) or items with an inter-item correlation between +/− 0.6, and negative scoring of marked areas outside the patella on the knee pain map (data not shown). Receiver operating characteristic (ROC) curves were derived to estimate the area under the curve (AUC) for sensitivity vs 1-specificity for each of the following comparisons: mean total score, section combinations and each section alone between the PFP group and the other two groups combined, between the PFP group and those with knee pain in the other two groups and between the PFP group and the soft-tissue injury group alone. The scoring method which gave the best AUC was then used to determine the optimal threshold to discriminate between PFP patients and other participants, that is, it gave the maximum score when sensitivity and specificity were added together. The optimal scoring method and the optimal threshold was then applied to the follow-up questionnaires and Cohen’s kappa (k) used to explore test-retest reliability for discriminating PFP from other groups, with approximate 95 % confidence intervals (using a bootstrapping approach). This method was also used to explore test-retest reliability for individual items. Intra-class correlation coefficients were used to explore the test-retest reliability of section scores. Measurement error, the error inherent in the measurement tool, was explored using Bland-Altman plots with 95 % limits of agreement and the standard error of measurement (SEM) was estimated.

### Study two

Having identified the best scoring system in study one, we applied this to the responses to the SNAPPS IV questionnaire in study two. The optimal threshold was then used to estimate the prevalence of PFP in the study population and to compare the prevalence rates between males and females and between those aged 39 years or less and those aged 40 years or more. Differences were explored using chi-square tests of significance. A *p* value <0.05 was considered statistically significant.

### Sample size

In study one, we aimed to recruit 35–40 participants per group. Thirty-five participants per group would enable the detection of pairwise between-group differences in SNAPPS total scores effect sizes of at least 1 with over 90 % power (based on a Bonferroni correction for three tests and using an independent-samples t-test with equal variances); and, having determined and applied the optimal threshold of SNAPPS total scores, 35 participants in each group would enable the estimation of sensitivity and specificity (relative to the soft tissue injury group) of at least 90 % to within +/−7.0 % with 95 % confidence. A total of approximately 110 participants providing data at both time-points would allow an estimation of k for test-retest reliability (agreement) to within ±0.15 with 95 % confidence providing k is at least 0.6 (‘substantial agreement’), according to the Landis and Koch classification [23], and the percentages of positive and negative item responses are each 50 % or k is at least 0.7 and the percentages of positive and negative item responses are each at least 30 %.

## Results

### Study one

A total of 84 participants were recruited to study one, of which 26 were in the PFP group, 30 in the soft-tissue group and 28 in the healthy group. The mean ages of each group were similar (Table 1). There were fewer female participants in the PFP group compared to the soft-tissue and healthy group. Of the 84 participants, 5 appeared to complete the first section incorrectly. One participant in the PFP group and two participants in the soft-tissue group stated that they had no knee pain or problems in the previous 12 months: two of the three stated they had seen the doctor about knee problems and all continued to complete the remainder of the questionnaire indicating problems. Two participants in the healthy group stated they had knee pain or problems in the previous 12 months but neither had been to see a doctor because of knee problems; these participants completed the other sections of the questionnaire. All five participants were scored as if they had knee problems. The responses to individual questions in the baseline questionnaire are shown in Table 1. There were only 2 missing items across 2 different questions and both occurred in the same participant (Table 1); these were given a score of 0 in further analysis assuming that the participant had no specific problems. For those who completed each item of the questionnaire, i.e., those with knee pain (*N* = 58), the inter-item correlations for section 3 ranged from–0.69 to 0.59 and for section 2 ranged from–0.21 to 0.47.

The mean scores for each section, combinations of sections and for all sections combined are shown in Table 1. The distribution of scores was significantly different across and between the 3 groups for section 2 (*F* = 134.8, *P* < 0.001) and for section 4 (*F* = 47.0 *P* < 0.001). For section 3, there was a significant difference between the healthy group and both the PFP and soft tissue injury group, but there was no difference between the PFP and the soft-tissue injury group (Table 1). When analysis was limited to the 4 items not related to PFP, there was a significant difference between the PFP and soft tissue injury groups but not between the healthy knee and PFP groups.

**Fig. 1:**
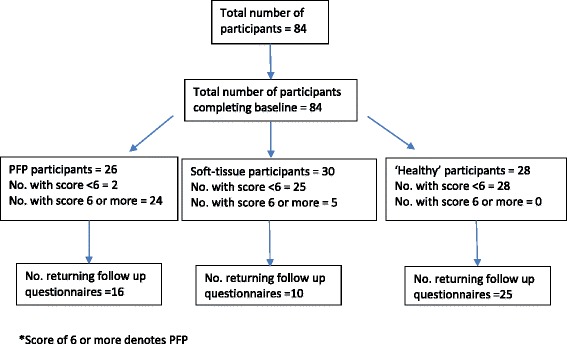
Flow chart for study one

The highest AUC was observed for the combination of section 2 (clinical features) and section 4 (knee pain map), suggesting that section 3 (activity-related pain) could be omitted from the questionnaire (Table 2). For this scoring system, the optimal threshold, with the highest sensitivity for the highest specificity, to discriminate between PFP and other groups was estimated to be an overall score of 6 or above for PFP. When this threshold was applied, the estimate of sensitivity was 92 % and the specificity was 94 %; 5 participants with other types of knee pain were misclassified as having PFP (Fig. 1). When the analysis was limited to those with just knee pain, the specificity was 84 %. There were 3 false positives when the threshold was increased to 7 but while the specificity increased to 91 %, the sensitivity fell to 77 %.

**Table 2:** AUC (95 % CI) for each questionnaire section for the baseline questionnaire estimated from ROC curves

	PFP group vs all others	PFP group vs all others with knee pain	PFP group vs soft tissue group
Section 2	0.94 (0.90 to 0.99)	0.90 (0.82 to 0.98)	0.91 (0.84 to 0.99)
Section 3	0.70 (0.58 to 0.81)	0.45 (0.30 to 0.61)	0.44 (0.29 to 0.53)
Section 4	0.92 (0.84 to 0.99)	0.88 (0.79 to 0.98)	0.88 (0.78 to 0.97)
Section 2 and 3	0.87 (0.79 to 0.95)	0.77 (0.64 to 0.90)	0.77 (0.64 to 0.90)
Section 2 and 4	0.97 (0.94 to 1.00)	0.95 (0.90 to 1.00)	0.95 (0.90 to 1.00)
Section 3 and 4	0.85 (0.77 to 0.93)	0.73 (0.59 to 0.88)	0.72 (0.58 to 0.87)
All sections	0.93 (0.87 to 0.99)	0.88 (0.78 to 0.98)	0.88 (0.78 to 0.98)

**Fig. 2:**
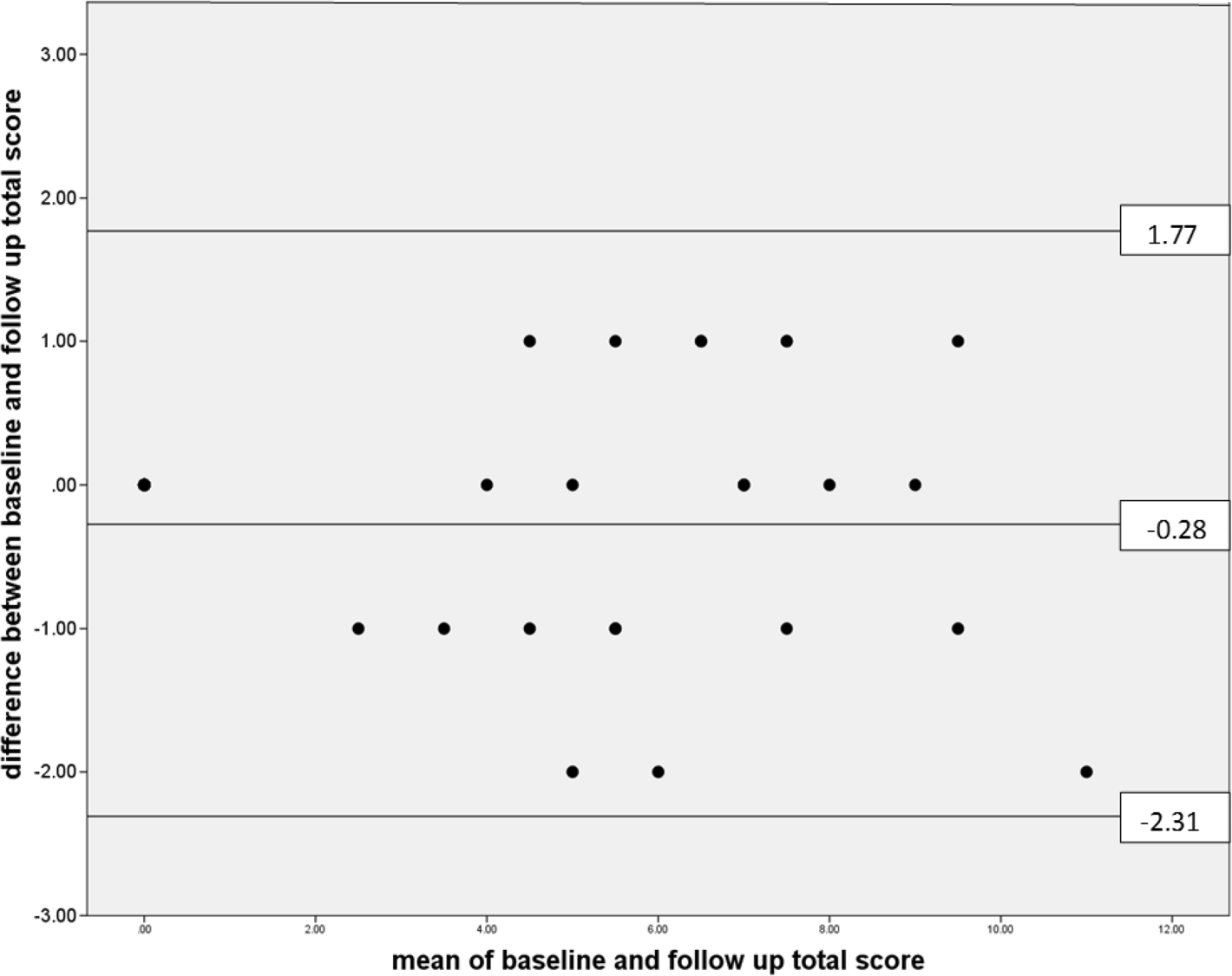
Bland Altman plot showing bias and 95 % limits of agreement (sections 2 and 4 combined)

A total of 51 (61 %) participants returned the follow-up questionnaires, of whom 16 were in the PFP group, 10 were in soft-tissue group and 25 were in the healthy knee group. After applying the same scoring method and threshold to the follow-up questionnaires, k was estimated to be 0.74 (95 % CI 0.52 to 0.91). The intra-class correlation coefficient for agreement was 0.97 (95 % CI 0.94 to 0.98) for section 2 scores and 0.79 (0.66 to 0.87) for section 4 scores. For 5 of the 7 items in section 2, k for individual items was greater than 0.69. However, it was 0.24 for the question ‘Have you had pain and discomfort for **more than one month**?’ and was 0.20 for the question ‘Thinking about your **right** (**left**) knee, what do you consider is your **main problem** with your knee?’ As the follow-up questionnaire was delivered two weeks after the baseline questionnaire, this could have resulted in more participants having pain for more than a month: six participants had pain for more than a month at follow up but not at baseline of whom 5 were in the soft-tissue injury group. When this question was excluded from the analysis, k rose to 0.84 (95 % CI 0.65 to 0.96). Figure 2 shows the Bland Altman plot for the final scoring method: the bias was–0.28 and the 95 % limits of agreement were–2.31 and +1.77. The SEM was estimated to be 0.74.

### Study two

There were 111 respondents: one participant who did not complete the knee pain map was excluded leaving 110 remaining participants for further analysis. The mean age was 30 years (SD 9.9) in the remaining 110 respondents and the ages ranged from 18 to 55 years. There were 91 (83 %) adults under 40 years of age. Information on gender was available for 109 participants: 58 (53 %) were female. The mean BMI for the 107 participants for which this was recorded was 25 kg/m^2^ (SD 4.4). The responses to individual items on the questionnaire and the mean scores for each questionnaire section and sections 2 and 4 combined are outlined in Table 1. A total of 38 participants (35 %) reported they had knee problems in the last 12 months: the prevalence of knee problems in those under 40 years of age was 33 % and in those over 40 years of age was 42 %. Of the 38 participants with knee pain, 25 (66 %) participants had a threshold score of 6 or above and would be classified as having PFP and, hence, using the questionnaire, the prevalence of PFP in the study population was 23 %. Of those with PFP, 16 (67 %) were female and 8 (33 %) were male. This was compared to 7 (50 %) and 7 (50 %) respectively of those with other knee problems; the difference in gender distribution between the groups was not statistically significant (chi square 0.45, df1, *P* = 0.50). Of the 30 participants with knee problems in those under 40 years of age, 20 (67 %) fulfilled the criteria for PFP, while among the 8 participants with knee problems who were over 40 years of age, 4 (50 %) fulfilled the criteria for PFP; this difference was not statistically significant (chi square 0.21, df1, *P* = 0.65).

## Discussion

As far as we are aware, this is the first attempt to devise a questionnaire which could discriminate between those who have PFP and those who do not within the community. We have followed guidance on investigating and reporting the properties of measurement tools [22, 24]. Our previous concern about whether activity-related questions could help to discriminate between those with and those without PFP appeared to be borne out by our findings: [14] there was no significant difference between those with PFP and those with soft-tissue injury when all activities were included and, perhaps unsurprisingly on reflection, there was no significant difference between those with PFP and those with healthy knees when section 3 was limited to the four activities which were not typically associated with PFP. Therefore, we could considerably shorten the questionnaire. There was little missing data. In this circumstance, we made an assumption that the participant had no specified problem, but as there was very little missing data, this assumption would need to be explored in future studies. Limiting the questionnaire scoring to clinical features and the knee pain map, the measurement properties as estimated from study one were mainly good: the AUC for the ROC curve was high; high sensitivity and specificity (in excess of 90 %) was achieved using a threshold score of 6 or above for PFP. Measurement error, estimated using Bland Altman 95 % limits of agreement, appeared to be satisfactory as these limits were less than the differences in the total mean (clinical features and knee pain map scores combined) observed between the three groups in study one. The estimate of test–retest reliability was within the range considered as substantial agreement by Landis and Koch (0.61 to 0.8) [23], and the lower 95 % confidence limit was within the range considered to demonstrate moderate agreement (0.41 to 0.6) [23]. However, as many of the soft-tissue injury patients were likely to have acute injuries, they may be more likely to have had pain for at least a month at the time of the follow up than they were at baseline. This was the case for 5 of the 10 soft-tissue injury respondents and excluding this question from the analysis of test-retest reliability increased the k to 0.84 within the ‘almost perfect’ agreement category according to the Landis and Koch classification [23], with the lower 95 % confidence interval within the substantial agreement category.

Using the questionnaire, the prevalence rate of PFP in the population sample (study two) was estimated to be 23 %. This is similar to the much cited figure of 25 % [25], which this has described over recent years as misleading as it is derived from those attending specialist sports clinics [1]. Some high quality data from specific populations do exist. In adolescents aged between 12 and 17 years the point prevalence is reported as 7 % [2]. These are younger populations than our population sample. In military populations, reported prevalence for males is 12 % and for females as 15 % [19]. This is slightly lower than our prevalence for those aged between 18 and 25 years of age of 17 %. The best estimate available for the general population in the UK is 59.6 per 10,000 for the age group 15–29 years and 42.5 per 10,000 for the 30–44 years age group, but these are based on primary care consultations, which are likely to be lower than community-based prevalence rates [15]. Furthermore, as previously highlighted, the authors raised issues relating to possible under-ascertainment of this condition because of how it is coded on primary care computer systems. The difference between our findings and primary care consultation rates raises concerns about possible unmet need in the community; although in these studies we did not collect information about pain levels. To some extent our prevalence rate may be inflated as the chosen threshold of 6 leads to a number of soft-tissue injuries being misclassified as PFP. A higher threshold of 7 appears to reduce the number of soft-tissue injuries being misclassified as PFP but would also lead to more of those with PFP being misclassified. Decisions about the most appropriate threshold to use will be dependent on the reason the questionnaire is being used. Clinical studies or natural history studies, in which it is important to be more sure about the diagnosis of PFP, may warrant using the higher threshold of 7.

### Study limitations

The number of recruited participants was lower than the á priori sample size and the low response rate led to less than half the target sample size for the estimation of k for test-retest reliability. Despite this the confidence intervals for k precluded slight or fair agreement (<0.41). The response rate for the follow-up questionnaire was particularly low amongst those who had a knee problem. Although it was possible to estimate test-retest reliability for just those with knee problems, the interpretation of this would be difficult given the number of respondents. However the questionnaire is designed for general populations, in whom we estimate the prevalence of knee problems to be around a third. The baseline questionnaire was administered after the participant was assessed by the physiotherapist. This may potentially introduce bias as the PFP inclusion criteria included questions about pain on activity. However, the section on pain on activity did not help to discriminate between the groups. We chose to repeat the questionnaire in two weeks in order to reduce recall bias but also to ensure the questionnaire was completed before treatment started. However, some of the soft-tissue injuries may have improved spontaneously by this time and this could have reduced the reliability. Despite this the test-retest reliability was still within the range considered substantial by Landis and Koch (0.61 to 0.8) [23].

In the general population study (study two), there were differences in age and gender distributions as expected but these known-group differences were not significantly different. The lack of statistical significance could be due to the sample size: given the opportunistic nature of the study, a pre-study power calculation was not undertaken. Further community-based studies are warranted. However, there were twice as many women classified as having PFP as men, which is in line with the gender differences reported in the literature [19]. Conversely in study one, there were more male participants recruited than female participants. It is not clear why this is, but has been observed occasionally in other PFP studies [26], and may be related to access to services or concerns about the problem. However, it did ensure that the demographic characteristics of the three groups in study one were more similar than we might have expected (Table 1).

More pertinent to population-based studies, another concern about the questionnaire is that other musculoskeletal disorders are being misclassified as PFP. For example, when scoring the knee pain map, we scored pain in the patella tendon the same way as pain in the medial or lateral patella. Pain solely located in the patella tendon may be more indicative of patella tendonitis than PFP. None of the participants with PFP only had pain in the patella tendon and all PFP patients had been clinically assessed as having pain in the medial and lateral facets of the patella. Further work is needed to explore the diagnostic accuracy of this questionnaire in community samples using prospective study designs. It may be prudent in these studies to include the section on activity-related pain (section 3), to examine whether it is useful in discriminating between PFP and other minor musculoskeletal knee disorders. However, given the difficulty of finding funding for validation studies, particularly for a condition which is still considered by many to be self-limiting, this may need to be undertaken during population-based prevalence, incidence, aetiological and/or natural history studies.

## Conclusion

We have developed a questionnaire which appears to have high sensitivity, specificity and test-retest reliability to discriminate between those with PFP and those who do not, based on clinical features and a knee pain map. This questionnaire needs to be further tested in community based studies to assess its discriminatory and test-retest measurement properties and in different countries to assess cross cultural validity. The full questionnaire is available in the Additional file 1.

## Abbreviations

AUC, area under the curve; CI, confidence interval; PFP, patellofemoral pain; ROC, receiver operating curve characteristic; SEM, standard error of measurement.

## Additional file

Additional file 1: SNAPPS–Survey instrument for Natural history, Aetiology and Prevalence of Patellofemoral pain Studies. (DOCX 111 kb)
