# Supplementary material for: A questionnaire to identify patellofemoral pain in the community: an exploration of measurement properties
Source: BMC Musculoskelet Disord. 2016 May 31;17:237. doi: 10.1186/s12891-016-1097-5 (PMC4886395; doi:10.1186/s12891-016-1097-5)
Supplement: Additional file 1: — SNAPPS–Survey instrument for Natural history, Aetiology and Prevalence of Patellofemoral pain Studies. (DOCX 111 kb) [file 12891_2016_1097_MOESM1_ESM.docx]

**SNAPPS- Survey instrument for Natural history, Aetiology and Prevalence of Patellofemoral pain Studies**

Q1.1 Are you aged over 18? Yes No

Q1.2 Are you aged under 40? Yes No

Q1.3 How old are you? Years

**If you are aged 18-40, please continue to Q2.**

Q2 Have you **ever** been to a doctor because of knee problems? (Please place a cross in one box only). Yes No

Q3 Have you had pain or problems in **the last year** in or around the knee? (Please place a cross in one box only).

Yes No

**If you have answered yes to Q3**, please continue to Q4.

**If you answered no to Q3**, please go to Q14.

Q4 In which knee have you had pain or problems? (Please place a cross in one box only)

Left knee only

Right knee only

Both knees

Q5 Have you had surgery to your knee? (Including arthroscopy, keyhole surgery, camera in your knee) (Please place a cross in one box only)

No

Yes, Left knee only

Yes, Right knee only

Yes, Both knees

Q6 Have you ever had a knee cap that has gone out of joint (dislocated)? (Please place a cross in one box only)

No

Yes, Left knee only

Yes, Right knee only

Yes, Both knees

Q7 Since starting with your knee problem, does your knee **ever swell up**? (Please place a cross in one box only)

No

Yes, Left knee only

Yes, Right knee only

Yes, Both knees

Q8 Have you had pain and discomfort for **more than one month**? (Please place a cross in one box only)

No

Yes, Left knee only

Yes, Right knee only

Yes, Both knees

Q9a Because of your knee problems would you suffer from pain or difficulty with **sitting for a long time**? (Please place a cross in one box only)

No

Yes, Left knee

Yes, Right knee

Yes, Both knees

Q9b Because of your knee problems would you suffer from pain or difficulty with **going up stairs**? (Please place a cross in one box only)

No

Yes, Left knee

Yes, Right knee

Yes, Both knees

Q9c Because of your knee problems would you suffer from pain or difficulty with **going downstairs**? (Please place a cross in one box only)

No

Yes, Left knee

Yes, Right knee

Yes, Both knees

Q9d Because of your knee problems would you suffer from pain or difficulty with **squatting**? (Please place a cross in one box only)

No

Yes, Left knee

Yes, Right knee

Yes, Both knees

Q9e Because of your knee problems would you suffer from pain or difficulty with **standing for long periods?** (Please place a cross in one box only)

No

Yes, Left knee

Yes, Right knee

Yes, Both knees

Q9f Because of your knee problems would you suffer from pain or difficulty with **walking on a level surface?** (Please place a cross in one box only)

No

Yes, Left knee

Yes, Right knee

Yes, Both knees

Q9g Because of your knee problems would you suffer from pain or difficulty with **getting up out of a chair?** (Please place a cross in one box only)

No

Yes, Left knee

Yes, Right knee

Yes, Both knees

Q9h Because of your knee problems would you suffer from pain or difficulty with **kneeling?** (Please place a cross in one box only)

No

Yes, Left knee

Yes, Right knee

Yes, Both knees

Q9i Because of your knee problems would you suffer from pain or difficulty with **walking on uneven surfaces?** (Please place a cross in one box only)

No

Yes, Left knee

Yes, Right knee

Yes, Both knees

Q9j Because of your knee problems would you suffer from pain or difficulty with **walking down slopes?** (Please place a cross in one box only)

No

Yes, Left knee

Yes, Right knee

Yes, Both knees

Q9k Because of your knee problems would you suffer from pain or difficulty with **walking up slopes?** (Please place a cross in one box only)

No

Yes, Left knee

Yes, Right knee

Yes, Both knees

Q9l Because of your knee problems would you suffer from pain or difficulty with **hopping**? (Please place a cross in one box only)

No

Yes, Left knee

Yes, Right knee

Yes, Both knees

Q9m Because of your knee problems would you suffer from pain or difficulty with **jumping**? (Please place a cross in one box only)

No

Yes, Left knee

Yes, Right knee

Yes, Both knees

Q9n Because of your knee problems would you suffer from pain or difficulty with **running**? (Please place a cross in one box only)

No

Yes, Left knee

Yes, Right knee

Yes, Both knees

We are now going to ask you some questions about each knee.

Starting with your **right** knee.

Q10a Thinking about your **right** knee, what do you consider is your **main problem** with your knee? (Please place a cross in one box only)

Pain or discomfort

Locking

Giving way or feeling like it will give way

No problem in this knee

Q10b Thinking about your **right** knee, did your current knee problem come on

(Please place a cross in one box only)

Because of a sudden injury e.g. twist, fall or accident that you needed to see a doctor about

Gradually over a period of time

Neither gradually nor because of a sudden injury

Not sure, can’t remember

No problem in this knee

Now we are going to ask you some questions about your **left** knee.

Q11a Thinking about your **left** knee, what do you consider your **main problem** with your knee? (Please place a cross in one box only)

Pain or discomfort

Locking

Giving way or feeling like it will give way

No problem in this knee

Q11b Thinking about your **left** knee, did your current knee problem come on

(Please place a cross in one box only)

Because of a sudden injury e.g. twist, fall or accident that you needed to see a doctor about

Gradually over a period of time

Neither gradually nor because of a sudden injury

Not sure, can’t remember

No problem in this knee

**Q 12 Please take a moment to think about where you get your knee pain.**

We would like you to imagine that this is a picture of your knees.

Please use small crosses to mark where you feel your knee pain on this

Diagram. You can use several crosses if needed.

www.photographickneepainmap.com

Q13 Considering both your knees which would you say is the knee that gives you most problems?

Always right

Usually right

Right and left equally

Usually left

Always left
